# Supplementary material for: What does it take to learn the rules of RNA base pairing? A lot less than you may think
Source: bioRxiv. 2025 Aug 2:2025.07.31.668042. Preprint. [Version 1] doi: 10.1101/2025.07.31.668042 (PMC12324431; doi:10.1101/2025.07.31.668042)
Supplement: Supplement 1 [file media-1.gz › supplemental_material/src/rscape_v2.6.0.tar.gz!/rscape_v2.6.0/lib/infernal/Manuscripts/inf-1-appnote/figs/gc.pdf]

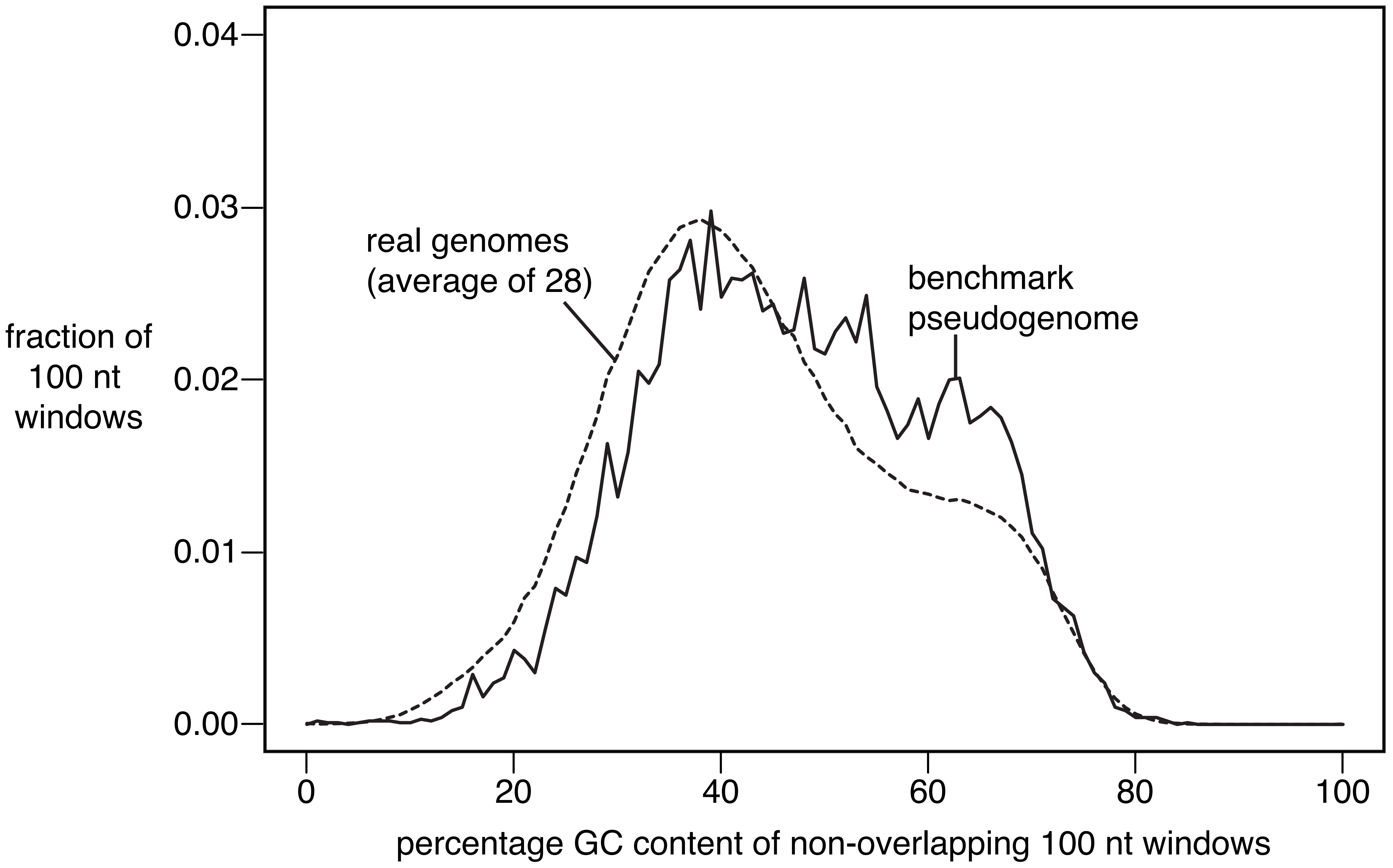

0.04
0.03
0.02
0.01
0.00
fraction of
100 nt
windows
real genomes
(average of 28)
benchmark
pseudogenome
0
20
40
60
80
100
percentage GC content of non-overlapping 100 nt windows
